# Rethinking Outsider Art in the digital age: an overview of Cara Macwilliam’s artistic practice

**DOI:** 10.1017/S2045796024000702

**Published:** 2024-11-13

**Authors:** Isil Ezgi Celik

**Affiliations:** CapitArt, Research and Curation, London, UK

## Outsider Art: a notion in flux

**Figure 1.**
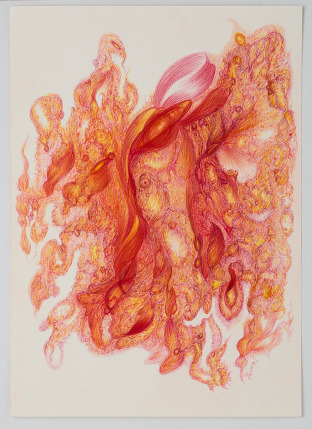
Cara Macwilliam, the fire licked the cave walls as they sang their songs of old, 2024, watercolour pencil on paper, 25 × 24cm, photo by Laura Hutchinson.

The concept of Outsider Art originates from Jean Dubuffet’s mid-20th-century notion of *l*’*Art Brut* (Raw Art), referring to art produced by individuals outside mainstream cultural institutions. Dubuffet (1949, trans. by the author), influenced by countercultural movements, criticised institutional constraints on creativity, seeking authentic, unconditioned expressions from creators detached from socio-cultural norms. However, despite Dubuffet’s attempt to position *l’Art Brut*’s authenticity on the cultural fringes, its recognition was paradoxically shaped by the very socio-cultural dynamics and values it sought to oppose. As a result, *l*’*Art Brut* has been the subject of prolific and often controversial conceptualisations, leading to decades of debate about its authenticity (Figure 1).

In the early 1970s, art critic Roger Cardinal introduced *l*’*Art Brut* to the English-speaking world as Outsider Art, shifting the focus from rawness to the marginalisation of its creators (Cardinal, 1972). This conceptual shift reflected broader socio-cultural changes, including the decline of countercultural movements, the rise of welfare states and digital global transformation. In the art scene, particularly with the Pop Art movement, the reproducibility of digital images played a central role in contesting traditional notions of artistic authenticity and undermined the power of institutional discourses on (high) art in an increasingly visually saturated global culture. Pop Art thus opened a space for the perception of Outsider Art (Rexer, 2005), coinciding with a shifting socio-political climate. The term has since expanded to include artworks from creators marginalised by evolving definitions of ‘outsider’, becoming one of the most ambiguous and contested terms in art history.

## Mainstream art and marginality in the digital age

**Figure 2.**
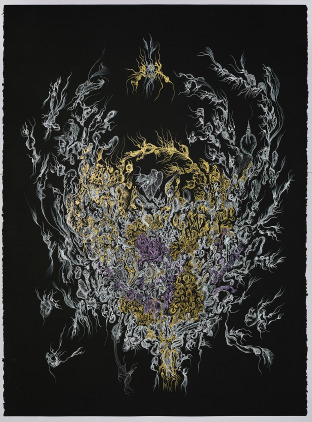
Cara Macwilliam, the holding of Artemisia’s dripping heart, 2024, dip pen and ink on vellum paper, 56 × 76 cm, photo by Laura Hutchinson.

Within the contemporary context, Colin Rhodes (2000) argued that the mainstreaming of Outsider Art – while validating marginalised artists – undermines its distinctive qualities. Others, such as Maclagan (2009), criticised the commodification of marginality within the mainstream, often evidenced by an overemphasis on the artist’s biography rather than their artwork. This commodification is not a recent phenomenon. Historically, Outsider Art derived its value from the perceived social marginality of its creators. Therefore, Outsider artists were excluded from formal art circles, while intermediaries commodified their works and biographies. Even Dubuffet had removed several creators from his *l*’*Art Brut* collection when they became artistically self-aware (Peiry, 2016, trans. by the author). Today, digitalisation offers artists greater chances to express their creations and speak for themselves, blurring traditional boundaries between insiders and outsiders in the art scene (Figure 2).

Marginality has always been fluid, shaped by socio-cultural norms (Çelik, 2018). Digital capitalism has not only redefined the boundaries of marginality but also of mainstream art. The rise of digital platforms has expanded access to artistic production, distribution, and appreciation, enabling artists to bypass traditional institutional gatekeepers. While this has meant greater inclusivity, it has simultaneously introduced new forms of exclusion. Unequal access to digital technologies continues to marginalise many, and algorithmic frameworks driven by market logic shape the visibility and reception of artworks, determining who is included or excluded.

**Figure 3.**
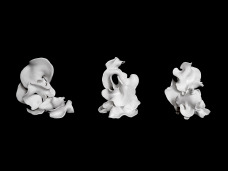
Cara Macwilliam, CaraMacwilliam, being becomes an agreement, 2024, polymer clay.

Exclusion mechanisms within the capitalist art market differ from those of traditional institutions. Institutions marginalise based on discourses of cultural legitimacy, deeming certain works central or peripheral based on predefined historical categories such as ‘authenticity’. In contrast, the capitalist market, while seemingly inclusive, tends to exclude anything that impedes capital flow, only to later reappropriate marginalised works as commodities within an expanding cycle of exchange (Deleuze, 1995). The market has thus eroded institutional authority over what constitutes artistic value, replacing it with a system governed by market logic. As Žižek (2004) argues, the homogenising logic of capitalism absorbs the very notion of ‘outside’ as a space for resistance or critical thought, making it increasingly difficult to differentiate and maintain an external position. Therefore the new mainstream, driven by the market and commodification, blurs the boundaries between marginality and the centre – absorbing Outsider Art, erasing its distinction from the outside and drawing it closer to the centre (Figure 3).

Outsider Art must therefore be reconsidered within this evolving socio-economic context. Defining Outsider Art by the creators’ separation from the mainstream has grown increasingly complex in today’s homogenised art world and the dilution of marginality cannot simply be attributed to artists’ self-awareness or increased visibility, as this overlooks the complex dynamics at play within the contemporary art scene. How, then, should we approach Outsider Art, today, when both mainstream art and marginality have fundamentally changed? Does it still – or did it ever – make sense to approach Outsider Art through rigid taxonomies, risking the oversimplification of the fluid nature of marginality within evolving socio-cultural dynamics? How can we resist the market’s reduction of marginality and Outsider Art to consumable products, while restoring the socio-political value of difference and marginality?

These are complex questions. However, given that the meaning of Outsider Art is shaped by audience perception, its socio-political significance necessitates a more nuanced engagement with Outsider artists and their work on their own terms, rather than forcing them into predefined frameworks or commodified narratives.

## Cara Macwilliam’s Outsider artistic practice

The artistic practice of self-identified Outsider artist Cara Macwilliam illustrates how digitalisation reshapes the boundaries and meanings of contemporary Outsider Art. Her artistic journey began after a transformative illness in early adulthood, which drastically reduced her independence and cognitive abilities. This rupture marked a shift from her previous intellectual life to one defined by marginalisation, and her practice emerged as a creative response to these limitations. Macwilliam (personal communication, 21 September 2024) acknowledges the controversy surrounding the term Outsider artist, particularly in the contemporary art scene, but she ‘strongly identifies with this nomenclature and classes its reappropriated use as a staunch political statement’.

**Figure 4.**
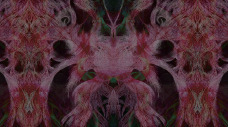
Cara Macwilliam, arisen from the forest by the strength of poor Yorick’s mind, 2022, digital.

Macwilliam, an entirely self-taught artist, works across various media – painting, drawing, textiles, sculpture and digital formats – employing mixed-media techniques. Her creations are deeply spiritual, informed by daily ritualistic practices. Her intuitive process manifests in a fluid expressiveness that transcends intentionality. Viewing all materials – whether physical or digital – as spiritual entities, she sees herself in symbiosis with unseen energies through which creative forces flow. Her practice expands into the digital realm. Guided by intuition, she selects and layers images instinctively to create video works allowing dynamic, fluid abstract compositions to surface. These video works act as extensions of her physical art works, with their fluidity translated into a mathematical universe, expressing a flowing existence that resists all limits. Her practice embodies a process of becoming, merging her life with her practice, experiencing creativity in spirituality, spirituality in nature, nature in humanity and humanity intechnology (Figure 4).

Macwilliam’s art celebrates the process of constant becoming through fluid, vibrant forms, with luminescent colours that diffuse into one another in a continuous flux of resonating energies – marked by the ease of movement she no longer physically experiences. Rooted in a profound sense of marginality, her work reappropriates the rupture that once derailed her life and places her outside a homogenising society obsessed with efficiency, into a flow of life that manifests the unity of multiplicities and differences.

In her work, every mark is integral – whether thumbprints, ink smudges, or digital glitches – dissolving the notion of mistake or imperfection. Her inclusive practice celebrates all that exists, free from valuation based on predetermined or learned categories. Her art becomes a creative response to societal exclusion and stigmatisation, actively inviting audiences to reflect on their subtle biases towards those consistently placed on the margins. Macwilliam’s art blurs the boundaries between the physical and the digital, the material and the immaterial, the inside and the outside.

## Epilogue

As digitalisation continues to reshape the boundaries of art, the distinction between mainstream and Outsider Art becomes increasingly indistinct. While digitalisation has enabled Outsider artists to access new platforms and reach wider audiences, it has also altered the nature of the mainstream, which is now driven by market logic and algorithmic control. This change complicates traditional evaluations of Outsider Art based on its distance from the mainstream and problematises the notion that Outsider Art lose value through self-awareness or visibility of its creator. The commodification of marginality is neither new nor unique to contemporary Outsider Art; it reflects broader capitalist dynamics shaping the art world and society. Artists like Cara Macwilliam, who gain visibility in the art scene through digitalisation and assert their own narratives of marginality, invite a reconsideration of what it means to be an outsider in the digital age. Rather than defining Outsider Art by its creators’ distance from the mainstream, it is crucial to understand the fluidity of marginality within evolving socio-political contexts. We should look closely at Outsider Art creations to appreciate the unique narratives of difference within each, rather than seeking similarities for categorical definition. The contemporary relevance of Outsider Art depends on the audience’s ability to reframe its political and creative edge, revealing exclusion and narratives of difference beyond generalised categories in an increasingly homogenised digital world.
